# Ready-to-Cook Foods: Technological Developments and Future Trends—A Systematic Review

**DOI:** 10.3390/foods13213454

**Published:** 2024-10-29

**Authors:** Tianqi Cui, Goh Rui Gine, Yuqin Lei, Zhiling Shi, Beichen Jiang, Yifan Yan, Hongchao Zhang

**Affiliations:** 1 College of Food Science and Nutritional Engineering, China Agriculture University, Beijing 100083, China; amberctq@163.com (T.C.);; 2 Key Laboratory of Fruit and Vegetable Processing of Ministry of Agriculture and Rural Affairs, Beijing Key Laboratory for Food Non-Thermal Processing, China Agriculture University, Beijing 100083, China

**Keywords:** ready-to-cook, fresh-cut, marination, modified atmosphere packaging, meal kits

## Abstract

Ready-to-cook (RTC) foods can significantly improve the cooking experience of busy or unskillful consumers, based on production involving technical combinations of food processing and packaging. Initialized by a market survey of 172 products in Beijing, this systematic review analyzes RTC foods’ development status according to ingredients, packaging, and storage conditions to further clarify the scope of RTC foods. The working principles and efficacy of various food processing techniques, such as washing, cutting, marinating, and frying, and packaging design or innovations such as modified atmosphere packaging (MAP) were both summarized in detail, with attention to their ability to extend shelf life, reduce safety risks, and maximize production efficiency in RTC food production. The cutting-edge technologies that may potentially apply in the RTC food processing or packaging sector were compared with current approaches to visualize the direction of future developments. In conclusion, we have observed the specific pattern of RTC food varieties and packaging formats in the Beijing market and revealed the advancements in RTC food technologies that will continue playing a critical role in shaping this growing market, while challenges in scalability, cost-efficiency, and sustainability remain key areas for future research. The data and perspectives presented will articulate the conceptions and existing challenges of RTC food, foster consumer perception and recognition of similar products, and deliver useful guidance for stakeholders interested in such products.

## 1. Introduction

**Figure 1:**
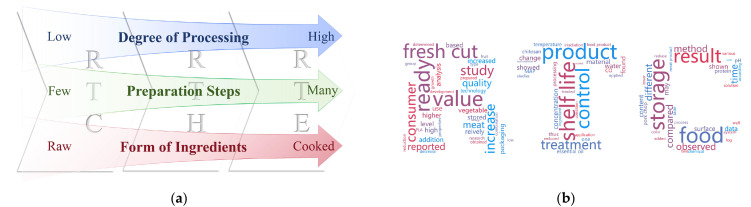
Conception of RTC foods: (**a**) Illustration of the differences in 3R foods regarding their degree of processing, levels of preparation, and ingredient conditions. (**b**) RTC word cloud map. After extracting the text content of a total of 78 texts in the literature related to RTC processing, the cloud map was generated using the WordCloud library for Python.

Ready-to-cook (RTC) foods are meals that have been pre-processed and are ready to be consumed with few or well-described cooking steps [1,2]. This type of food is favored by consumers for its convenience and freshness [3]. The main feature of RTC food is the ability to prepare delicious dishes in a relatively short period of time through simple cooking, by using ovens, microwaves, pans, etc., to meet the modern consumer’s demand for fast and healthy eating [4,5]. In the 1980s, Professor Masayuki Yoshikawa of Japan proposed the concept of “3R food”, i.e., “ready-to-eat”, “ready-to-heat” and “ready-to-cook” [6,7,8]. The origin of 3R food was the HMR (home meal replacement) concept in the United States. In the 1960s, the proliferation of standardized meal enterprises in the U.S. gave a direct impetus to the industrialization of prepared dishes. In the 1980s, prepared food expanded to Japan, gradually developing from supermarkets and convenience stores to catering companies, etc. In Japan, prepared food is referred to as “Nakanoshoku”, a third form of dining between eating out and home cooking. Today, advanced global supply chains and rapid lifestyle changes have accelerated the diversity and global reach of 3R foods, and the market for RTC food is growing rapidly [6,8,9]. A comparison between 3R foods and a word cloud of concepts relevant to RTC food are displayed in Figure 1.

The global RTC market has seen significant expansion, with the market size projected to grow at a compound annual growth rate (CAGR) of 6.83% from 2019 to 2025, approximate valuing $156.8 billion by end of 2025 [10]. According to *Market.us*, North America holds the largest share of the market, commanding 41.0%, followed by Europe (24.0%), the Asia-Pacific region (20.0%), and the rest of the world (15%) [10]. China, as an emerging market and a technology-driven market, is fueled by urbanization and increasing disposable incomes. Prepared foods which encompasses RTC products in China is projected to exceed USD 144.8 billion by 2026, with an average annual growth rate of 25% [9,11], reflecting a substantial growth trajectory and a significant potential for RTC products.

Despite the extensive interests and significant revenue in the development of RTC meals, the industry also faces some challenges, such as the lack of uniform industry standards [9], widespread consumer concerns about food safety and health attributes [12,13], as well as the retention of sensory quality which is the persistent issue that needs to be overcome. A successful RTC product must possess good overall quality close to the fresh ingredients when it reaches the consumers, with satisfactory sensory attributes after cooking, while also having manageable costs for both the manufacturers and consumers. Therefore, the advancement of RTC product development requires a high level of technical solutions [9], from the farm to the table, particularly for optimized levels of food processing and packaging with targeted technologies.

Typically, RTC foods need to be pretreated to remove dirt, microbial loads, etc., prior to removal of unused parts and processing into desirable shapes or cuts. Optionally, further processing can be applied, such as marination, frying, fermentation, etc., depending on the ingredients and requirements of dishes before cooking. Owing to the perishable nature of most RTC foods, antimicrobial approaches, packaging, and cold chains are necessary for maximizing the shelf life [14,15,16]. Currently, cleaning and sanitation are often deficient in maintaining freshness or may even increase the risk of microbial contamination [17]; traditional cutting and peeling techniques are prone to high energy/labor consumption and mass losses [18]; other processes such marination, frying, and sanitation also need to be improved in their effectiveness, feasibility, and sustainability [19]. Despite progress in RTC food development, it has not been recognized by most consumers; there is also a need for specialized information to guide researchers and industry professionals in identifying, standardizing, and advancing the subcategories of RTC foods, with niche technologies for improving each of them. Therefore, this review aims to fill this gap by providing a systematic analysis of existing technologies, highlighting areas that require further research, and offering insights into potential innovations that could drive the RTC sector forward. With growing consumer demand for healthier and higher quality products, emerging technologies such as irradiation [7], high-speed cutting [20], high-pressure processing (HPP) marination [21], and modified atmosphere packaging (MAP) exhibit excellent potential to solve the existing problems and meeting the consumer’s increasing expectation; hence, they are the key to the RTC industry’s continued growth and competitiveness.

This systematic review focuses on products, processing, and packaging of RTC foods, with in-depth details covering the development status and cutting-edge progress. To our knowledge, although RTE foods have been extensively studied and reviewed in recent years, there are no reviews focusing on RTC foods. Through systematic sorting and analysis, this paper expects to provide theoretical references and practical guidance for the consumer recognition and industrial innovation of RTC foods.

## 2. Methods

This systematic review was performed in three steps: conducting the search, reviewing abstracts and full texts, and discussing the results. The review involved a comprehensive and systematic approach that also included market surveys and visualization techniques. For the literature review, databases such as ScienceDirect, Web of Science, and Google Scholar were searched to identify relevant studies aligned with the objectives of the review. The final search was conducted in September 2024, including scientific published articles, online reports, and book chapters. The keyword “ready-to-cook food” was used for searching in combination with other terms such as “prepared food”, “modified atmosphere packaging” “meal kits” “consumer perception” or “market trends”. After completing the search, all duplicates were removed, and the abstracts of the remaining articles were carefully reviewed to ensure they met the review’s inclusion criteria, which covered studies focused on ready-to-cook foods, particularly in areas such as ingredient preparation, processing technologies, packaging, and market trends. Studies fitting these criteria were then synthesized to form the basis of this systematic review. Additionally, data from a market survey were integrated to enrich the review with empirical evidence, and visualizations such as pie charts and graphs were used to enhance the clarity and presentation of the findings.

**Figure 2:**
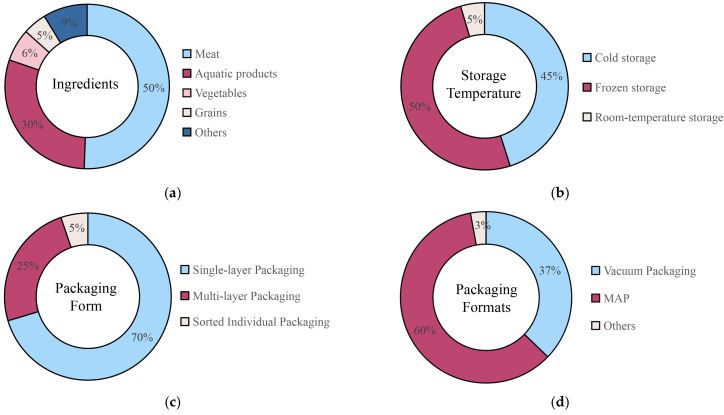
Statistics of RTC foods in the Chinese market, based on a survey of local supermarkets in Beijing: (**a**) RTC classification statistics based on ingredients; (**b**) statistics on storage temperature; (**c**) packing methods or technologies; (**d**) overall packaging formats.

As shown in Figure 2, a survey of 172 RTC products from two supermarkets in Beijing was used to analyze the existing RTC product formats in market. The selection of products was based on packaging claiming that the product is “ready-to-cook” or if the product’s status matched the “uncooked” and “prepared” definition provided in this review. All products fitting these criteria were included rather than a random selection. In addition to that, the pie charts provide an analysis of the categorization of RTC products by their major ingredient, storage temperature condition, and packaging used. Although the survey cannot reflect the situation in other countries or regions or represent the entire Chinese market due to significant urban/rural differences, it does offer useful reference data for large cities in China and can illustrate the trends in other Asian urban markets with relatively high-end consumer segments.

## 3. Product Formats of RTC Foods with a Market Survey as a Reference

As shown in Figure 2, meat, aquatic products, vegetables, rice, noodles, cereals, and more are among the RTC foods studied in this survey. Of these foods, meat and aquatic products constitute 80.2% of the Chinese market, higher than the 50% RTC market share reported by developed countries [22,23,24]. The popular products in this category were reported to be RTC kebabs, marinated steaks, fish fillets, and squid rings [3,4,25,26]. There are also some unique products such as frozen lamb or beef rolls pre-portioned for hotpot use. In comparison, France has the second largest prepared meal market in Western Europe, with 38% of RTE or RTC dishes being meat-based [27,28], and a similar trend is seen in the UK, where 86% of UK adults eat prepared meals, with meat and seafood products having a market share of 66–87% among major retailers, with 61% of ready-to-eat meat products being beef and chicken, and lamb and fish products have the fastest-growing market shares, with 12% and 11% annual growth rates from 2017 to 2022, respectively [29,30]. The rich variety of meat products in China, including detailed subdivisions such as pork, beef, lamb, and poultry, provides consumers with a wide array of choices, contributing to the overall market dominance of meat-based RTC products [31]. This data reflects a preference for meat-based RTC products in both China and Western countries, driven by consumer demand for convenience and familiar flavors. The meat-heavy structure of China’s RTC food market reflects the deeply ingrained cultural importance of meat consumption, which is driven by both traditional dietary preferences and increasing disposable income levels [32].

In terms of storage temperature, freezing dominates at 50.6%, particularly in meat and fish. Frozen RTC products enjoy high popularity due to their long shelf life and compatibility with the fast-paced urban lifestyle, especially in metropolitan areas where cold chain logistics have developed significantly in recent years [31]. Market consisted of steady share of frozen RTC products possibly due to the advantages of long shelf life compatible for almost all products. Meanwhile, chilled products also boomed, with a 45.3% market share based on the survey data, indicating that products with shorter but fresher shelf life, such as pre-marinated slices of roasted meat gained more popularity than ever before [25]. Previous study revealed that U.S. market demonstrated a lower prevalence of chilled RTC products, with only 36% of stores offering such products, which contrasts with the higher availability of frozen products across 63.6% of stores surveyed [33]. Based on the fast development of cold-chain facilities in China, quick freezing and warehouse storage of RTC products are no longer a limitation, at least in urban areas.

As far as packaging methods are concerned, modified atmosphere packaging and vacuum packaging are the predominant choices, both of which can significantly extend shelf life while maintaining freshness and sensory quality. Our survey data show that modified atmosphere packaging is particularly popular among meat RTC products in the Beijing market, accounting for 59.9% of packaging choices [34]. At the same time, although 70.3% of packages are still in a single-layer form, they tended to comprise more functionalities, particularly for their convenience and cooking process indicators on the packaging itself. Nevertheless, only 61.0% of the survey products state the cooking methods or provide cooking instructions in details, possibly because these products are in simple and flexible format to be used in various dishes, rather than a well-designed meal [35,36]. This trend is also observed in a study of the Spanish market, where RTC food packaging is largely focused on maintaining shelf life, though with less emphasis on detailed cooking instructions, which are only provided for 55% of such products [37]. As for materials, plastic remains the most common outer packaging material, including plastic boxes, pouches, and vacuum-sealed bags. Cartons also account for a small portion [4,26,34], whereas more complex multiple individual bags are used more and more frequently for packing precise quantities of each ingredient as individual packs inside a meal box.

In summary, survey results not only reveal the diversity and development trend of RTC foods in the Beijing market but also provide an important reference for future product development.

## 4. Food Processing Technologies for RTC Food Product

### 4.1. Washing, Sanitation, and Antimicrobial Interventions

**Figure 3:**
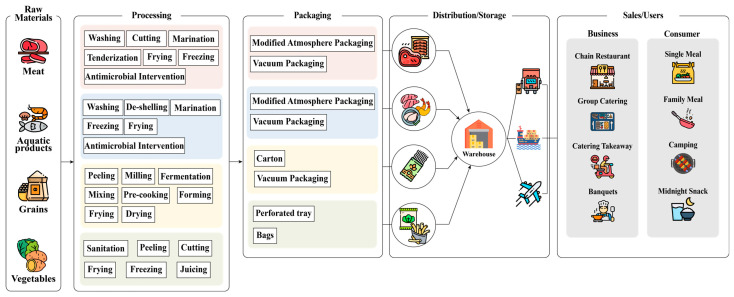
Sketch of RTC food supply chain.

Due to the nature of RTC foods, most of them need to be initially washed and sanitized to remove debris and microbial load, then pre-cooled for the cold chain [38], as shown in Figure 3. They also need to be treated with antimicrobials along with the washing or by adding antimicrobial components inside the package, i.e., antimicrobial interventions (essential oil [39], organic acid [40], chitosan [41], etc.), in order to maximize the shelf life of the unsterilized ingredients; in addition, pathogenic contamination needs to be prevented.

Most RTC foods are assembled from washed raw materials, they require different levels of cleaning. Particularly, the processing of fresh produce into RTC involves three essential water washing stages: primary washing, washing and sanitizing, and rinsing [42,43]. The primary washing serves to eliminate large impurities, such as soil particles, insect fragments, etc., from unpeeled vegetables, while the cleaning and sanitizing phase is critical for minimizing microbial attachment and enhancing shelf life. The final rinsing step is designed to remove any residual detergent [42]. Meanwhile, for meat carcasses, washing after skinning and before gutting is useful to reduce microbial adhesion [44], in addition to washing at the end of slaughter and before freezing or refrigerated processing as an important step in decontamination [45], e.g., washing in hot and cold water, lactic acid decontamination. Fresh seafood exhibits a high degree of perishability following harvest, necessitating immediate processing, which includes cleaning, trimming, peeling, and gutting [46]. For instance, oysters require thorough washing with water to remove any mud, followed by prompt shucking, which can be achieved through methods such as steaming or infrared heating [47]. Similarly, lobsters should be meticulously cleaned without delay, taking care to shield them from sunlight and direct winds, before being subjected to freezing for preservation [48].

In the washing processes of these raw materials, in addition to large particles of impurities, reducing microbial load is another key task. Spoilage microorganisms can easily proliferate on food matrix rich in nutrients, which are heavily exposed to the environment post-processing [49,50]. Meanwhile, fresh produce and meat carcasses may harbor pathogenic bacteria such as *Escherichia coli*, *Salmonella*, *Staphylococcus aureus*, *Campylobacter* spp., and *Listeria monocytogenes* [45,51,52]. While seafood products are of particular concern due to the presence of *Vibrio* spp. [53] and *L*. *monocytogenes* [54]. Contamination with both spoilage and pathogenic microorganisms may occur at any point of the processing or even during the packaging and distribution, necessitating urgent cleaning and intervention measures [52,55,56]. Therefore, water washing works as a multi-purpose approach that can handle the above-mentioned issues within a single process. The related technologies can be divided into physical and chemical, but in most cases, they are used in combination with each other to reach the optimized efficiency [15,57].

The most frequently used chemical approach for sanitizing RTC foods is adding chlorine-based sanitizers when washing them [58]. Needless to say, chlorine-based disinfectants are effective in reducing initial microbial loads; the commonly used disinfectant sodium hypochlorite (NaOCl) has strong oxidizing properties and is bactericidal against a wide range of microorganisms such as bacteria, viruses, fungi, and protozoa. However, the effectiveness of chlorine relies heavily on the wash water quality [17], resulting in unreliable antimicrobial effects. Other chemical sanitizers developed for washing and sanitation include chlorine dioxide, PAA, ozone, and electrolyzed water [58]. These novel sanitizers have not been widely applied by the food industry, although their antimicrobial efficacy was demonstrated [59,60]. For instance, Al-Holy and Rasco (2015) [59] applied acidic electrolyzed water on trout, chicken, and beef muscle for 10 min, causing, respectively, reductions of 1.5, 1.5, and 1.4 log in *Salmonella Typhimurium* and reductions of 1.2, 1.1, and 1.3 log in *L*. *monocytogenes*. The main concerns are the increased cost and safety risks in a large-scale production scenario [61,62,63,64].

Normally used physical techniques include turbulence- and bubble-assisted washing, which are extensively employed to improve mass transfer [65,66]. Novel ultrasound- or microbubble/nanobubble-assisted washing methods have also been investigated with increasing interest in recent years [67,68]. The cavitation and shearing effects of ultrasound or bubbles can inactivate or scrub microorganisms through mechanisms of cavitation and shear forces without leaving any dead space, and help to maintain the quality characteristics of agricultural products [69,70], especially fragile leafy vegetables. However, the implement of these technologies for food cleaning is currently limited to a laboratory scale and has not yet been adopted for commercial food products [71,72]. By optimizing the cleaning conditions for single-frequency ultrasound using response surfaces, Alenyorege et al. (2020) [73] found that the removal of 5.6 and 4.7 CFU/g for *E. coli* and *L. innocua* respectively could be achieved by washing Chinese cabbage for 15 min using ultrasound with a frequency of 40 kHz and a power of 125.45 W/L. Ozone micro- and nanobubbles with an ORP of 860 ± 42 mV were sufficient to reduce *Streptococcus agalactiae* or *Aeromonas veronii* concentrations by 26- to 48-fold, corresponding to a reduction exceeding 96%, when applied to tilapia products for 10 min [67]. These new techniques overall demonstrated significantly improved cleaning and sanitation efficacy, particularly when applied simultaneously with chemical sanitizers [66,74].

In other cases, some RTC foods are not suitable to be washed or sanitized. Irradiation is an alternative way for microbial mitigation, without compromise in food quality. Gunes et al. (2011) [7] treated ground beef patties with different doses of irradiation and found that *L*. *monocytogenes* was effectively inactivated at a dose of 3 kGy, whereas *E*. *coli* was inactivated at a lower dose of 1.5 kGy. Furthermore, the shelf life of the ground beef product was extended to 21 days when stored at a temperature of 3 °C. The application of ultraviolet light (UV-C) light-emitting diodes (LEDs) at wavelengths of 250–280 nm for the disinfection of skinless chicken breasts contaminated with *Salmonella enterica* yielded a reduction of 1.02 and 1.78 Log CFU/cm^2^ after exposure for 1 min and 15 min, respectively [75]. Irradiation technologies, including ultraviolet (UV) light, are currently employed in commercial applications due to their low cost and absence of residuals, but mainly in liquid food products [76]. Similarly, other techniques such as high-pressure processing also demonstrated effective application in antimicrobial intervention of RTC seafoods. Kural et al. (2008)’s [77] research indicated that applying a pressure of at least 350 MPa for 2 min at temperatures ranging from 1 to 35 °C, or a pressure of at least 300 MPa for 2 min at 40 °C, resulted in a reduction of 5 log in the inactivation of *Vibrio parahaemolyticus* in oysters. A summary of representative studies with novel antimicrobial intervention technologies for RTC foods are listed in Table 1.

In the case of solely antimicrobial intervention, many food grade compounds demonstrated the suitability when directly added into the food or its brine inside packages. for example, essential oils (e.g., rosemary oil and thyme oil) exhibit broad-spectrum antimicrobial effects by disrupting bacterial cell membranes and are commonly used to prolong the shelf life of meat and vegetable RTC foods [78,79]. Nisin, a naturally occurring antimicrobial peptide produced by lactobacillus bacteria, is able to inhibit the growth of gram-positive bacteria, and is particularly suited to controlling *Listeria monocytogenes* in meat products [80].

**Table 1:** Representative cleaning, sanitation, or antimicrobial techniques used for pathogen control in RTC foods.

RTC Food	Techniques	Treatment Conditions	Effectiveness	References
Washed vegetables	Electrolyzed water	50 ppm of free chlorine, 45 s	4 log CFU/g *Salmonella* inactivation	[81]
Tomato beef brisket	Peroxyacetic acid	10 mg/L, 30 s	Prevent cross-contamination with 10^6^ log CFU/g *Salmonella*	[60]
Chicken skewer	Peroxyacetic acid	0.07%, 15 s	2.0 log CFU/mL reduction in aerobic bacteria and *Salmonella*	[82]
Thick-cut grilled meat slices	Chlorine dioxide	200 ppm/400 ppm, 30 s	0.73/1.25 log CFU/g *E. coli* O157:H7 inactivation	[83]
Trout fillet	Acidic electrolyzed water	pH 2.30, free chlorine: 38 ppm, 10 min	1.5 log *Salmonella Typhimuriu* and 1.2 log *L. monocytogenes* reduction	[59]
Korean Army stew	Ultrasound	40 kHz, 125.45 W/L ultrasound power, 15 min	5.6 and 4.7 log CFU/g for *E. coli* and *L. innocua* reduction, respectively	[73]
Washed vegetables	Ultrasound	40 kHz, 100 W/L, 1 min	2.5 and 2.6 log CFU/g for *E. coli* and *L. innocua* reduction respectively	[84]
Beef patty	Gamma irradiation	3 kGy/1.5 kGy	totally inactivating *L. innocua* and *E. coli* respectively	[77]
Riced cauliflower	Irradiation	0.5 kGy	2 log CFU/g inactivation of total aerobic bacteria	[5]
Spicy crayfish	High-pressure processing	≥350 MPa at 1–35 °C or ≥300 MPa at 40 °C, 2 min	5.0 log cfu/g *Vibrio parahaemolyticus* inactivation	[77]
Clean tilapia fillets	Peroxyacetic acid	300 ppm, fogging, 15 min	1.66 CFU/g *Salmonella* reduction	[85]
Hairtail fish balls	High-pressure processing	300 MPa, 5 min	707.67 CFU/g total colony reduction	[86]

### 4.2. Peeling- and Cutting-Related Technologies

RTC foods post initial cleaning are submitted to subsequent steps such as peeling of fresh produce, skinning of meat, shell removal from shellfish, and cutting. By converting the raw materials to nearly 100% edible portion, these processes greatly reduce the preparation time for consumers during cooking and may assist them in completing complex and delicate food preparation [47]. Currently, for many RTC foods, these procedures are still completely or partially reliant on manual work, while some others are already performed with machinery [87].

Fresh produce RTC foods, such as potatoes and tomatoes, require peeling before further processing. Traditionally, peeling of fresh produce often involves methods such as lye peeling and steam peeling; these methods are efficient but have drawbacks such as high energy consumption, high peeling losses, and environmental concerns [18]. In recent years, researchers have been trying out new peeling techniques for fruits and vegetables, such as ohmic heating [18,88], infrared peeling [89], and ultrasonic peeling [18]. These techniques are less intense thus posing minimal impact on product quality and will be suitable for future fresh produce RTC foods. Skinning of meats relies more on physical separation by specially designed equipment; large-volume processing also requires a standard supply of animals whose physical properties, such as dimensions, are compatible with the processing line [90,91]. In the category of RTC seafood, some fish products only need to have their scales removed; however, shell removal from shrimp and oysters is more challenging and still heavily relies on manual work in small-scale processing. In recent years, researchers have found that high-pressure technology can be applied to the peeling of shellfish. For example, Xuan et al. (2018) [92] have used high-pressure technology to achieve peeling of fresh razor clams; the shelling rate reached up to 100% for a holding time of 10 min at a pressure of 200 MPa or 1 min at a pressure of 400 MPa.

For the cutting step, high-speed cutting technology, which predominantly utilizes a mechanical cutting mode, is the most prevalent method employed in food processing facilities. The technology has been used in the processing of RTC meat, vegetables, and dairy products. However, higher cutting accuracy has been demanded [93] in order to prevent substantial food residue generation [94]. For example, when dealing with separation of specific part of animals or fish, such as steaks or fish heads, it is essential to achieve precise cutting to obtain the exact portion. More innovative cutting technologies have emerged, including ultrasonic vibration-assisted cutting, laser cutting, and water jet cutting. However, ultrasonic vibration-assisted cutting is deemed inappropriate for the processing of vegetables and meat due to the significant enzymatic reactions that occur in the effluent, as well as the potential for thermal damage [95]. Laser cutting and water jet cutting are classified as non-contact cutting methods. Laser cutting is a thermal cutting technique that involves directing a concentrated laser beam onto the material’s surface, resulting in rapid localized heating that facilitates fine structural separation; this makes laser cutting more appropriate for customized processing applications [96]. However, this method is not suitable for materials that are sensitive to heat. In contrast, water jet cutting is a non-thermal technique that utilizes the substantial kinetic energy of a high-speed water jet to impact and separate the material [97]. This approach also supports custom processing and can be combined with advanced technologies such as computer vision, making it particularly appropriate for soft materials in the processing of RTC foods, such as vegetables. For meat cutting, high-pressure water jets may be a better choice; they are able to cut bones with a higher level of cutting force than normal water jets [98]. These advanced methods offer enhanced precision and quality in cutting processes, thereby providing safer and more efficient alternatives. Additionally, some of the food materials need to be smashed, powdered, or even reconstituted to maximize the flavor or provide a more pleasant mouthfeel when used in RTC foods [99,100,101].

### 4.3. Marination Technology

Marination is a pivotal process in the preparation of ready-to-cook meals, as it substantially enhances the organoleptic properties, including both taste profile and mouthfeel [19,102]. During the traditional marination, foods are soaked in a marinade, e.g., salt solution, soy sauce, or customized sauce, over several hours or overnight. This is effective for infusing flavor but is less efficient for RTC meals that require quicker processing times. Furthermore, traditional marination can result in uneven flavor penetration, especially in larger or denser items [103]. Typically, marination times of 12 to 24 h are needed for adequate flavor absorption in vegetables and small cuts of meat or fish. Traditional marination is cost-effective, requiring only basic kitchen tools or industrial vats, which are significantly cheaper compared to advanced technologies [19].

In contrast, newer techniques with commercial applications include vacuum marination, injection marination, and tumblers [104,105,106]. Vacuum marination accelerates this process by reducing air and enhancing the penetration of flavors, while injection marination quickly infuses flavors into large volumes of product, making it efficient for high-throughput operations. Tumblers and rotating drums provide uniform flavor distribution and tenderness through mechanical agitation, which is ideal for bulk processing. More specifically, vacuum marination uses a vacuum chamber to remove air, creating a pressure differential that accelerates marinade absorption. J. and M. (2023) [107] highlight that this method reduces marination times from 12 h to 1–2 h for meats and poultry. However, the cost of vacuum marination equipment varies widely, with smaller machines costing between $2000 and $5000, while industrial systems such as the BLENDTEC 3500 lb tumbler can cost more than ten times as much [108]. This method is particularly effective for RTC meals due to its ability to enhance the penetration of key flavor components quickly and uniformly. Vacuum marination works by creating a low-pressure environment that expands the food’s cellular structure, allowing marinade components such as sodium, spices, and acids to diffuse more rapidly and deeply into the food. This is facilitated by the removal of air pockets and the creation of a pressure gradient that drives the marinade into the food matrix. Optimizing parameters such as vacuum pressure (≥400 MPa) and marination duration can further improve the uniformity of flavor and seasoning, ensuring that critical components reach the deeper parts of the food efficiently. Injection marination involves directly injecting marinade into food using needles or pumps, allowing rapid flavor infusion within 15 to 30 min for large cuts of meat or poultry [104,109]. This method is highly effective for RTC meals where uniform flavor distribution is essential. The cost of commercial meat injectors ranges from $5000 to $50,000 depending on automation and capacity [110]. However, careful control of injection parameters, such as pressure and volume, is required to prevent texture changes. Injection marination is beneficial for large or dense items that need quick marination before cooking. Tumblers or rotating drums continuously tumble food with the marinade, processing it within 30 min to 2 h, depending on the product and desired flavor level [111]. Tumblers vary in cost, with smaller models available for as little as $6500, while larger industrial models can reach up to $35,000 [112]. The key parameters include tumbling speed and drum rotation time, which ensure even coating and flavor distribution.

Emerging technologies primarily studied in laboratory settings include high-pressure processing (HPP), ultrasound marination, and electro-magnetic fields (EMF) [113,114]. HPP applies high pressure to enhance marinade absorption, achieving uniform flavor infusion in just a few minutes while preserving food texture. However, HPP has certain limitations that hinder its broader application. For instance, HPP-induced lipid oxidation, baroresistance of bacterial spores, and changes in food color are some significant challenges of this technology [115]. Moreover, HPP equipment is relative expensive, ranging from $500,000 to $2.5 million [112]. Pressures of 400–600 MPa applied for 3 to 5 min have been shown to provide superior marinade penetration and preservation of food texture. HPP is ideal for RTC meals such as marinated meats and seafood, where rapid flavor infusion and high-quality preservation are critical [116,117]. Although HPP provides superior marinade penetration and texture preservation, pressures of 400–600 MPa applied for 3 to 5 min may not fully inactivate bacterial spores, requiring supplementary techniques such as cold-chain handling for safety [118]. Ultrasound marination uses high-frequency sound waves to accelerate the marination process, reducing times to 20–40 min. Costs for ultrasound systems range from $20,000 to $100,000, depending on capacity and system sophistication [108]. This method is effective for various food categories, including meats and vegetables, where faster marination is advantageous. The technology involves optimizing frequency and power settings to enhance absorption rates while maintaining food quality [107]. EMF technology employs electromagnetic fields to improve marinade absorption, though it remains an emerging and costly technology with potential benefits for rapid and uniform flavor distribution. Zhang et al. (2023) [78] indicate that EMF can enhance flavor penetration effectively, though it is still in the experimental stage and is comparable in cost to ultrasound marination [108]. The method shows potential for a wide range of food categories, including meats and vegetables, with the ability to achieve uniform marinade distribution rapidly.

Each marination technology offers unique advantages based on factors such as efficiency, processing scale, and food categories. Their application scope and specific characteristics are listed in Table 2.

**Table 2:** Comparisons among marination technologies used for RTC foods.

Technology	Molecular Mechanism	Marination Efficiency	Food Categories	References
Traditional Marination	Flavor molecules move via simple diffusion from higher to lower concentrations. Outer layers absorb most flavor; center less affected.	Relatively slow (12–24 h).	Vegetables, small cuts of meat, fish	[103,119]
Vacuum Marination	Reduced air pressure in vacuum chamber speeds up marinade diffusion, removing air pockets and enhancing infiltration into food spaces.	High; faster than traditional (1–2 h).	Meats, poultry, fish	[103,105,107,120]
Injection Marination	Marinade penetrates food interior uniformly through injection channels, distributing salts, sugars, and flavors evenly.	High; precise and fast (15–30 min).	Large cuts of meat, dense products	[104,112,121,122]
Tumblers/Rotating Drums	Constant tumbling increases contact between marinade and food, facilitating uniform and rapid marinade absorption.	High; effective for large batches (30 min to 2 h).	Meats, poultry, seafood	[106,111,123,124]
High-Pressure Processing (HPP)	High pressure disrupts cell membranes, enhancing food matrix permeability for deeper, uniform marinade penetration.	Very high; rapid and deep penetration (3–5 min).	RTC meals, high-value products	[21,120,125,126]
Ultrasound Marination	Ultrasonic waves generate cavitation, causing micro-shocks and turbulence that mix marinade and enhance flavor molecule absorption.	High; faster absorption (20–40 min).	Meats, seafood, vegetables	[107,127,128,129]
Electro-Magnetic Fields (EMF)	EMF technology interacts with the food matrix, influencing marinade component movement and absorption.	Potentially high; still developing.	Meats and vegetables	[93,113]

### 4.4. Frying Technology

Frying is another important process to achieve a unique flavor and desirable texture in RTC foods. Common frying technologies in the current market include deep-fat frying, vacuum frying, and air frying. Traditional deep-fat frying refers to immersing food in high-temperature oil and utilizing heat conduction to rapidly dehydrate the surface of the food and form a crispy outer shell. It is widely used for products such as meat and aquatic products, in which, by virtue of its high efficiency and rapidity, it results in a crispy outer surface and a tender inner surface, but it can easily lead to a high fat intake and pose a health risk [130,131]. Vacuum frying is suitable for the production of dried vegetables, which can retain more nutrients and natural color under low-temperature and low-pressure environments, but the high cost restrained its applications in RTC vegetables such as carrot crisps [132]. Due to the configuration of frying process inside a vacuum chamber, such processes are essentially of the batch type and have limited throughput. Even a large piece of equipment 12 m long and 3 m wide can only produce about 350 kg of product per hour, and the equipment is relatively expensive [131]. Air frying uses circulating hot air instead of fat to significantly reduce fat content, in line with the trend of healthier diets, but differs from traditional deep frying in terms of taste [130,133,134].

Frontiers in frying technology studied for RTC applications focused on the coupling technology of vacuum frying with microwave, ultrasound, or radio frequency in a targeted manner according to product characteristics to obtain higher-quality pre-fried food [133,135]. Microwave-assisted vacuum frying (MVF) integrates microwave heating within a vacuum environment by uniformly positioning microwave generators around the frying chamber. This technology leverages microwave energy to enhance the heating and water evaporation of food products, significantly reducing the fat content of food products while improving the color and crunch, and it is suitable for the production of high-quality RTC vegetables, meats, and aquatic products [133,136,137,138]. In studies conducted by Su et al. (2016), Su et al. (2018), Zhang et al. (2020) and R. et al. (2021), microwave power levels of 600 W to 1000 W were used, with frying times of up to 360 s. This approach significantly reduces fat content, with one study showing a decrease from 39.14 g/100 g to 29.35 g/100 g during the frying of potato chips [136,137,139]. While MVF allows for the production of crunchy food products and maintains the basic quality of the product, the process requires longer processing times, resulting in higher operating costs and investments [140]. Pulse-spouted microwave vacuum frying (PSMVF) uses intermittent pulses during the frying process instead of continuously spraying oil onto the food. This pulse allows for more precise control of the amount and coverage of oil, reducing oil consumption and optimizing thermal efficiency through intermittent heat input, enabling precise control of the process. This is particularly beneficial for RTC root vegetables with high starch content, such as potatoes, because the method efficiently manages the moisture content critical to maintaining texture and flavor after frying [137,141]. In a study by Su et al. (2018), pulse-jet microwave technology combined with vacuum frying at 90 °C was shown to reduce fat content by 16–34% [142], while maintaining the desired color [143,144,145]. However, research on PSMVF is still at the laboratory stage with no commercial usage yet. Ultrasonic microwave-assisted vacuum frying (USMVF), on the other hand, introduces ultrasonic waves on the basis of MVF, which enhances the rate of water evaporation through the cavitation effect, reduces the absorption of oil and fat, and causes more nutrients and color to be retained. The USMVF is suitable for the efficient processing of various RTC foods. For example, in the processing of purple potato chips, USMVF not only shortens the frying time but also significantly improves the retention of anthocyanins [139]. This is particularly advantageous for producing heathier RTC fried foods since high temperature frying can hardly improve the retention of heat-sensitive components, which are usually important portion of the nutrients. Studies by Su et al. (2018), Islam et al. (2019), and Zhang et al. (2020), at ultrasound power levels of 300 W to 600 W, coupled with microwave power of 600 W to 800 W, shortened frying time while improving the retention of anthocyanins and nutrients. The cavitation effect from ultrasound accelerates moisture removal, improving texture and crispness while reducing oil content by 16–34% [133,142,146]. The technology is particularly effective at lower frying temperatures, making it suitable for items with sensitive nutritional components that might degrade at higher heat. This method shows a promising reduction in drying time by 20–28%, and the enhanced moisture evaporation rates lead to improved product crispness and reduced oil content [139,147]. The integration of ultrasound technology into MVF is also cost-effective, as the equipment costs about 1/10 of the total cost of the frying setup. The energy used by the ultrasound is significantly less than that used by the microwave source and vacuum pump, reducing overall energy consumption. This makes USMVF an economically viable alternative that enhances the quality attributes of fried products at lower temperatures, suggesting that combining ultrasound with MVF could be a beneficial technique for producing high-quality fried products efficiently [139].

Additionally, capabilities for reheating or re-frying of pre-fried RTC foods are important consideration, involves using ovens, air fryers, or microwaves to restore the original texture and flavor, bringing the food closer to a freshly prepared state [148,149]. It has been shown that the physical structure of the food significantly affects the amount of fat absorbed during the re-frying of frozen pre-fried foods, such as frozen French fries and battered fish fillets, and that lipid intercalation is evident. The use of different types of oils during deep-frying produced differences in the major fatty acid content of the foods and showed less preferential adsorption of polar compounds on the food surface [150,151]. By optimizing the breading formulation and frying parameters, microwave-heatable pre-fried foods can effectively maintain crispiness after re-frying, but large-scale economic production still faces challenges [152,153]. In the case of beef skewers, for example, the refrying conditions of 190 °C and 60 s can significantly improve their yield, color and sensory quality [154].

## 5. Novel Packaging Technologies for RTC Food

### 5.1. Novel Packaging Design for RTC Foods

**Figure 4:**
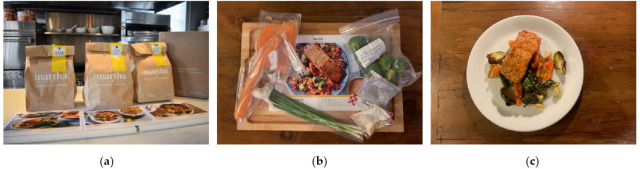
Meal kit images and recipes: (**a**) Outer packaging of a branded meal kit product. (**b**) Inner packaging for a branded meal kit brand product. (**c**) Finished product prepared from a branded meal kit product (source: https://www.themarthablog.com/2022/10/a-delicious-meal-from-martha-stewart-marley-spoon.html (accessed on 13 September 2024)).

As with other convenient foods, packaging for RTC foods is generally fabricated with polymer-based flexible materials or polymeric material inner packs with paper-based outside boxes, which was reflected in our survey. However, they certainly can be designed to achieve more functionalities. One of the most representative case is the meal kits, consisted of individual packs pre-portioned into resealable pouches or airtight containers to ensure freshness. Well-designed meal kits include special sizes and are made of small packages for dry spices, liquid sauces, and separate containers for individual protein sources or vegetables. In this way, to greatly avoid spills and cross-contaminations, and provide easy identification and organization for inexperienced users [155]. A typical meal kit product is showed in Figure 4 as below.

A user-friendly interface is also essential for a smooth and enjoyable cooking experience. Clear labeling of ingredients, nutritional information, and allergens is crucial for effective meal preparation and dietary management. Recipe cards with clear, step-by-step instructions and visuals guide users through the cooking process, detailing cooking times, techniques, and serving suggestions to ensure successful meal outcomes. This together with the ingredient organization enhances meal preparation efficiency and improves the overall user experience [155,156]. Recipe cards with clear, step-by-step instructions, visuals, and additional interactive features such as QR codes linking to online recipe videos or resources can enhance the overall user experience while also focusing on reducing the amount of paper and ink used [157,158,159]. The invention of meal kits successfully meets consumer demand for fresh ingredients and healthy eating while providing convenience and time savings. As shown in Table 3, meal kit products are commercially available in many companies in different countries and regions across the world. With evolving consumer preferences and the increasing diversification of market demand, the variety of meal kits will become even more abundant, covering different eating habits and cultural backgrounds, thus further driving the market expansion of RTC food.

Another example involving packaging design for RTC foods is compatibility with microwave ovens. For example, foods can be cooked by microwave oven in the original package with pores for outgoing steam, or metal board susceptors can be used to develop browning and crispness in pizza or pie [160]. Regarding packaging shape, rectangular boxes are commonly used for practicality in organizing and storing various components. Flexible stand-up pouches and stackable containers not only maximize space efficiency but also contribute to an appealing presentation. The choice of shape affects packaging functionality and consumer perception, impacting both usability and marketing effectiveness [155]. In addition to functionality, sustainability is a key consideration for RTC packaging. Efforts to reduce the carbon footprint of packaging include transitioning to recyclable and reusable materials, optimizing logistics to enhance transport efficiency, and reducing the volume and weight of packaging materials. For example, the use of flexible stand-up pouches with their reduced packaging and reusability helps to improve transportation and storage efficiency, ultimately reducing the environmental impact of RTC packaging [161]. More sustainable options are such as recyclable paper-based materials and lightweight plastic alternatives, which are also highly recommended [162,163]. This trend focuses on reducing the carbon footprint and the volume of plastic used in both single-layer and multi-layer forms. Packaging using fiber-based solutions combined with biodegradable coatings is being evaluated to enhance sustainability [163].

### 5.2. Modified Atmosphere Packaging (MAP) and Vacuum Packaging (VP)

Modified atmosphere packaging (MAP) and vacuum packaging (VP) are two major techniques used in RTC foods, with evidenced results of quality retention through the microbial inhibition and oxidation prevention, particularly useful for seafood and meat products [47,164,165].

MAP significantly extend the shelf life of RTC foods. The shelf life of Vietnamese *Pangasius hypophthalmus* fillets [166] packaged in air and vacuum was estimated to be 7 and 10 days. However, MAP (50% CO_2_–50% O_2_) increased the shelf life by 100%, bringing it to 14 days. Similarly, salmon fillets packaged in CO_2_: N_2_ (60:40) had more reddish and yellowish color than vacuum packaging and air, indicating a significantly longer shelf life. With MAP, fillets were stored at 4 °C for around 18–20 days with a limit of 106 CFU/g. Therefore, MAP effectively extended the microbiological shelf life of raw fillets by 1.5 times [167]. Especially, Benyathiar et al. (2020) has compared the shelf life of fresh-cut asparagus packaging under modified atmosphere packaging (MAP)and vacuum skin packaging (VSP) respectively [168]. VSP is a relatively new technique derived from traditional vacuum packaging for fresh and premium food options; it encloses the product like a blanket, and the product is secured tightly by simultaneously heating the transparent upper barrier film, then sealing the bottom of the tray [169]. It has been reported that both techniques, combined with refrigeration, help to maintain the freshness and product shelf life up to 21 days for MAP and 18 days for VSP. For chicken product, Dogu-Baykut and Gunes (2014) found that lactic acid bacteria in RTC marinated chicken drumsticks increased from 2.9 log CFU/g to 4.36 in MAP package (5% O_2_), while increased to 5.1 and 6.5 log CFU/g in vacuum and aerobic packages after 25 days [170]. The result demonstrated MAP could decrease these quality reduction. Nevertheless, studies have shown that vacuum packaging is also able to significantly bypass common packaging for shelf life extension, considering about the cost, VP is even more used compared with MAP. Overall, MAP and VP not only provide alternative solutions granting a longer shelf life than that of regular packed RTC foods but also present other advantages over traditional preservation. For instances, frozen storage mitigates the rate of microbial proliferation and chemical deterioration of seafood and meat [171]; however, the process of thawing and reheating is time-consuming, with high risks of microbial contamination and inevitable quality degradation by ice crystals [172,173]. The shelf-life of sea bass stored in ice, as determined by overall acceptability sensory scores and microbiological data, was 8–9 days for filleted and 12–13 days for whole ungutted fish [174]. MAP may also reduce the addition of antimicrobials or other substances and allow easy separation and good presentation of the contained products, as reported by other studies [84,175,176].

As listed in Table 4, gas compositions are critical for MAP-packed RTC foods. For vegetable and fruits, only small portion of O_2_ and CO_2_ would be sufficient to maintain the color and inhibit spoilage, without disturbing their respiration activities [177]. In contrast, for meat and poultry, O_2_ is necessary for a desirable red color, N_2_ is also required to prevent oxidation, and CO_2_ may be used for inhibiting microbial growth [178]; therefore, the overall condition is more complex. Additionally, a changed gas ratio could help to control some microorganisms that pose safety concerns, such as *Clostridium perfringens*, *Clostridium botulinum*, and *L. monocytogenes* [179].

However, it is important to note that MAP packaging has a higher cost compared to vacuum packaging and aerobic packaging, primarily due to the need for specialized gas mixtures and more complex sealing technologies [180]. Furthermore, MAP can increase the risk of anaerobic bacterial growth if not stored under controlled temperatures, as elevated storage temperatures could lead to the growth of anaerobic spoilage and pathogenic microorganisms such as *Clostridium botulinum* and *Listeria monocytogenes* [181]. Therefore, more research is required to reduce the costs of the materials and processing operations used to create intelligent packaging materials in the future so as to make them more commercially viable [182].

**Table 4:** Application of different types of corresponding air-conditioning ratios in RTC.

Food Category	RTC Food	O_2_ (%)	CO_2_ (%)	N_2_ (%)	Storage Temperature (°C)	Shelf Life (Days)	References
Seafood	Headed and filleted chub mackerel, yellow gurnard, hake fishes	5	95	0	4	14	[183]
	Deskinned and filleted tilapia	10	60	30	4	15	[184]
	Deskinned and filleted cape hake fish	30	40	30	0	12	[185]
	Whole gutted farmed bass	30	50	40	3	7–9	[186]
	Shucked and pasteurized oyster	/	75	25	0	24	[187]
Meat and poultry	Skinless chicken breast	/	40	60	4	6	[188]
	Raw beef meatball (ground beef, onion, bread crumb, black pepper, red pepper, cumin, salt, garlic)	3	50	47	4	21	[7]
	Boneless chicken breast	/	50	50	4	14	[189]
Vegetable and fruit	Cut and salted Chinese cabbage	0	25	75	4	21	[190]
	Broccoli heads were cut into florets	5	10	/	5	12	[191]
	Fresh, whole asparagus	21	0.03	/	4	21	[168]
	Fenugreek sterilized with sodium hypochlorite	10–14	5–8	/	8	15	[192]
	Papayas peeled and cut in half to scrape off the seed and the layer of flesh	7.2	5.2	/	15	6	[193]

### 5.3. Active Packaging

Innovative packaging technologies have been applied to RTC foods such as active packaging technologies include oxygen/water absorbers, antimicrobial packaging, and active and edible coatings [194,195,196]. These technologies aim to enhance product shelf life and ensure food safety by interacting with the food product or its environment. In recent years, the development of novel packaging systems has also gained attention, incorporating active elements that interact with the environment to maintain product quality.

Oxygen/water absorbers, for example, are already widely used in seafood and meat products, with brands such as Blue Apron, Gousto, and YouFoodz utilizing these absorbers in the form of small sachets or composite pads, often placed underneath the meat for direct contact [197]. These absorbers help reduce spoilage and extend the product’s shelf life. Meanwhile, antimicrobial packaging and edible coatings hold great potential but face challenges related to safety and efficacy when migrating into food matrices. There are ongoing debates about their mode of action, but their clean-label appeal—offering natural alternatives to man-made preservatives—continues to draw interest [198,199]. These innovations are paving the way for smart labels, which not only protect food but also enhance consumer trust through real-time quality feedback.

### 5.4. Intelligent Packaging

Intelligent packaging technologies, such as time–temperature indicators (TTI), printable RFID chips, and freshness sensors, provide real-time data on product quality during distribution and storage [195,200,201,202]. These technologies focus on tracking and communicating the status of the food, especially important for fresh and cold-chain commodities, including many RTC products. The incorporation of smart labels, such as freshness and spoilage indicators, is becoming more prevalent, providing consumers with an additional layer of quality assurance [203,204].

For instance, time–temperature indicators (TTIs) are among the most representative examples showing realistic applications specially for cold-chain fresh commodities. Regardless of the varying underlying principles, these indicators could vividly display the real-time freshness of consumers, through recording the time-temperature history during the food distribution and storage [205]. This is certainly important for RTC foods, as many of them have limited shelf life, and the freshness as presented to consumers during store display is critical for consumer decision. Some famous time–temperature indicators such as Timestrip^®^ and Keep-it allow visualized tracking of shelf life with minimal modification of the original package. Future trends in RTC packaging also include integrating active and smart packaging elements with recyclable or compostable materials to meet both consumer convenience and environmental sustainability needs [161]. Representative commercial active or smart packaging products potential for RTC applications are listed in Table 5 [12,195,206,207].

### 5.5. Biodegradable Packaging

Biodegradable packaging has emerged as another promising solution for RTC foods, addressing the growing need for environmentally friendly alternatives to conventional plastics. These materials, including bioplastics such as PLA and PHA [159], are designed to decompose naturally, reducing environmental pollution and reliance on fossil fuels. Recent innovations also include biobased edible coatings that enhance product safety while offering clean-label appeal [157].

Biodegradable materials are being integrated into packaging systems to balance food protection with sustainability [161]. For example, biopolymers such as cellulose are used to provide effective moisture and gas barriers, though challenges remain in achieving mechanical properties and cost efficiency comparable to those of traditional plastics [157]. Nonetheless, biodegradable packaging continues to draw interest due to its potential to align with circular economy principles and consumer demand for sustainability.

## 6. Future Perspectives

### 6.1. Consumer Acceptance

Consumer acceptance is crucial for the future development of RTC foods. The facts of RTC foods are not clear to many consumers; as convenience foods, they are often misjudged as unhealthy, unsustainable, and ultra-processed [9,11]. Although some RTC products have found success, their consumers are generally specific groups of people, leaving a great deal of large potential for market growth. In another aspect, due to a lack of technologies and equipment, RTC development in different countries and regions exhibit imbalance, resulting in distinguished qualities for similar products. To attract more consumers to these relatively new forms of food products, the industry needs to continue educating the public, promoting the products, improving product quality, and strengthening food safety management, with continuous product and technique development as support [208].

### 6.2. Personalized Food

Personalized nutrition is an emerging trend connecting with future developments in RTC foods [6,195]. With heightened health consciousness and the consumer’s pursuit for tailored dietary solutions, RTC foods are uniquely positioned to cater to individual nutritional requirements, preferences, and lifestyle choices. Research indicates that personalized dietary solutions, when effectively integrated with RTC offerings, might lead to enhanced customer satisfaction and better health outcomes. For example, personalized meal kits allow consumers to select ingredients and recipes based on their health goals, reflecting an increasing trend toward individualized nutrition [209]. By leveraging expertise from dietitians, advancements in data analytics, and artificial intelligence, RTC foods can offer customized meal solutions that align with specific health conditions and dietary needs [13,199]. This personalization enhances customer satisfaction, fostering a deeper connection between consumers and food brands. The results of recent studies have demonstrated that personalized food choice advice, tailored to individual socio-demographic and sensory preferences, significantly enhances consumers’ willingness to adopt healthier diets compared to generic advice [210]. This approach fosters a deeper connection between consumers and food brands while also offering companies opportunities to differentiate their products through unique, consumer-specific offerings [209]. Furthermore, it opens up new avenues for growth within the food industry, as companies can differentiate their offerings by providing unique, consumer-specific products. Meal kits, which allow consumers to select ingredients and recipes aligned with their dietary goals, exemplify this trend. Future advancements in RTC food personalization will likely involve a combination of genetic, phenotypic, and lifestyle data, allowing for even more precise nutritional solutions [209].

### 6.3. Distribution and E-Commerce

The growth of distribution and e-commerce along with the cold chain logistics has significantly bolstered the popularity of RTC foods [211]. With the widespread adoption of online shopping and advances in logistics technology, consumers now enjoy more convenient access to RTC foods. In China, the proliferation of fresh food e-commerce platforms has been particularly notable. Platforms such as *Hema Fresh*, *Dingdong Maicai*, *Meituan*, and *JD Daojia* have made it incredibly easy for consumers to order meal kits and other RTC foods. These apps not only provide a broad range of options but also ensure quick delivery directly to consumers’ doorsteps.

Looking ahead, delivery services for RTC foods are poised to become even faster and more efficient. The ongoing improvements in cold chain logistics technology will further enhance the freshness and safety of the delivered foods. These advancements are expected to drive even greater consumer adoption, as the convenience of ordering via mobile apps combines with assured food quality, meeting the increasing demand for quick, healthy, and safe dining options at home.

Another future to the RTC market would be the introduction to meal kits in China and other developing countries. As discussed above, meal kits not only consist of individual packed fresh ingredients but also include detailed instructions in various formats, facilitating consumers’ need of high quality meal in shorter time. Moreover, delivery methods for meal kits advance the convenience and freshness. Direct-to-door delivery offers scheduled times and subscription models for consistent meal planning, while click-and-collect services enable online ordering with pickup at retail locations or secure lockers. Courier services provide both standard and express delivery options, and temperature control measures, such as insulated packaging and eco-friendly solutions, maintain freshness throughout transit [155]. In the next 5–10 years, packaging innovations in RTC foods are expected to improve convenience for users and significantly reduce waste, thereby enhancing the sustainability of production. These advancements will likely focus on environmentally friendly materials and smart packaging solutions, contributing to a more sustainable food industry. Flexible delivery options further allow for customizable times and address adjustments to meet customer needs, collectively enhancing customer satisfaction by ensuring timely and optimal delivery of meal kits [155,158].

### 6.4. Emerging Technologies

With the fast growing of food processing and packaging technologies, new innovations will continuously show up for the future development of RTC food, with some of them possessing excellent potential for commercial applications. Examples are such as ultra-high-pressure processing [21,212], superheated steam [213,214], smart packaging [200,211], digital cold chain technology [211], each of which may provide niche application for improving quality and safety of RTC foods. Internet of Things (IoT) and blockchain technology will further advance the cold chain logistics, greatly improve the traceability, safety, and cost-efficiency during RTC food production [211]. It can be envisioned that the application of emerging technologies will also expand the application scenarios of RTC foods.

In summary, RTC foods will usher in multiple opportunities and challenges in the future market development. Through product innovation, personalized customization, logistics and distribution optimization, and the application of emerging technologies to enhance consumer acceptance, RTC foods may continue to maintain strong growth momentum and become an important part of modern dietary life.

## 7. Conclusions

In conclusion, the RTC food industry has experienced significant growth, driven by increasing consumer demand for convenience and healthier eating options. Our review highlights advancements in key processing and packaging technologies. Nevertheless, there is still a long road ahead for commercial applications of emerging technologies, considering cost–benefit analysis, consumer safety, and sustainability. Moreover, consumer perception and awareness will continue to shape the future of RTC foods, necessitating industry efforts to produce new RTC foods with high quality and low safety risks. The dataset size of our survey and the inaccessibility of information from practical production may limit our ability to fully capture the operational challenges in real industrial settings, highlighting the need for future research with a wider scope of investigation and detailed industry collaborations.

## Figures and Tables

**Table 3:** Meal kits and their product descriptions in different regions of the world.

Region		Meals Type	Packaging	Sources
North America (US)	Blue Apron	Diverse meal options, including classic, vegetarian, and wellness-focused recipes.	Cardboard boxes, recyclable packaging, ice packs.	https://www.blueapron.com, accessed on: 31 August 2024
	Sunbasket^®^	Organic and clean ingredient options with choices such as paleo, keto, and vegetarian.	Cardboard boxes, insulated liners, ice packs.	https://sunbasket.com, accessed on: 31 August 2024
	Green Chef	USDA-certified organic meals with various diet plans such as keto, paleo, and balanced.	Cardboard boxes, compostable or recyclable packaging, ice packs.	https://www.greenchef.com, accessed on: 31 August 2024
	EveryPlate	Budget-friendly meal options with straightforward recipes.	Cardboard boxes, recyclable materials, ice packs.	https://www.everyplate.com, accessed on: 31 August 2024
	Freshly	Fully prepared meals that are ready to heat and eat.	Plastic containers, cardboard shipping boxes.	https://www.bonappetit.com/story/freshly-review-meal-delivery-service, accessed on: 31 August 2024
	Gobble	Meals designed for quick preparation with pre-prepped ingredients.	Cardboard boxes, insulated liners, ice packs.	https://www.gobble.com, accessed on: 31 August 2024
	Snap Kitchen	Fully prepared, healthy meals focusing on balanced nutrition.	Plastic containers, insulated boxes, ice packs.	https://www.snapkitchen.com, accessed on: 31 August 2024
Europe (UK, Germany)	Gousto	Wide range of recipes including family meals, vegetarian, and calorie-controlled options.	Cardboard boxes, insulated liners, ice packs.	https://www.gousto.co.uk, accessed on: 31 August 2024
	Kochhaus	German-style meal kits with diverse recipes, including seasonal and regional dishes.	Cardboard boxes, recyclable or compostable materials, ice packs.	https://www.gessato.com/map_listing/kochhaus/, accessed on: 31 August 2024
	Chefkoch Box	German meal kits with a variety of recipes tailored to different tastes.	Cardboard boxes, recyclable materials, ice packs.	https://www.chefkoch.de/rs/s0/etepetete+box/Rezepte.html, accessed on: 31 August 2024
	Feast Box	Diverse meal options with a focus on high-quality ingredients and global cuisines.	Cardboard boxes, insulated liners, ice packs.	https://feastbox.co.in, accessed on: 31 August 2024
	Marley Spoon	Martha Stewart-inspired recipes with a variety of meal options.	Cardboard boxes, insulated liners, ice packs.	https://www.marleyspoon.com, accessed on: 31 August 2024
	HelloFresh^®^	Variety of recipes including classic, vegetarian, and family-friendly options.	Cardboard boxes, recyclable and compostable materials, refrigerated gel packs.	https://www.hellofresh.com, accessed on: 31 August 2024
Asia pacific (Australia, Hong Kong, Singapore)	YouFoodz	Ready-made meals, including options such as high-protein, low-calorie, and vegetarian.	Meals are packaged using Modified Atmosphere Packaging (MAP), sealed in recyclable plastic trays with cardboard sleeves.	https://www.youfoodz.com, accessed on: 31 August 2024
	Lite n’ Easy	Ready-made meals and meal plans designed for weight loss and healthy living, offering calorie-controlled and balanced options.	Meals are vacuum-sealed in plastic trays and delivered in insulated boxes to maintain freshness.	https://www.liteneasy.com.au, accessed on: 31 August 2024
	CookUp	Gourmet, pre-prepared meals crafted by chefs, offering a range of cuisines and dietary options.	Typically delivered in eco-friendly, microwave-safe containers made from recyclable materials.	https://www.cookupclasses.com, accessed on: 31 August 2024
	Box Green	Plant-based and vegan meal kits, emphasizing sustainable and healthy eating.	Uses biodegradable and compostable materials for both meal containers and delivery boxes.	https://www.boxgreen.com, accessed on: 31 August 2024
	The Good Kitchen	Healthy, balanced, chef-prepared ready-to-eat meals.	Meals are delivered in microwave-safe, recyclable containers that keep food fresh without preservatives.	https://www.thegoodkitchen.com, accessed on: 31 August 2024
Middle East and Africa (UAE, Saudi Arabia, South Africa)	Hello Chef	Varied cuisines, including vegetarian and low-carb.	Insulated, recyclable boxes with biodegradable packaging.	https://hellochef.me, accessed on: 31 August 2024
	Munchbox	Portion-controlled, healthy snacks, and meals.	Eco-friendly, portion-controlled containers.	https://www.mymunchbox.com.au, accessed on: 31 August 2024
	Afresh	Balanced, low-carb, and vegetarian options.	Eco-friendly, insulated, and recyclable materials.	https://www.afresh.com, accessed on: 31 August 2024
	Daily Dish	Vegetarian, gluten-free, and low-carb.	Recyclable containers designed for freshness.	https://dailydish.com, accessed on: 31 August 2024

**Table 5:** Representative commercial active or smart packaging products with potential for RTC applications according to region.

Product Name	Company	Function and Application	Sources
Freshness Sensors	Insignia Technologies (North Lanarkshire, Scotland)	Time–temperature indicators that alert about freshness, monitoring perishable RTC items.	https://www.insigniatechnologies.com, accessed on 10 September 2024
Oxygen Scavengers	Multisorb Technologies (New York, NY, USA)	Reduces oxygen in packaging to extend shelf life, crucial for preventing oxidation in meat, fish, and poultry.	https://www.multisorb.com, accessed on 10 September 2024
FreshTag	Cryolog (Paris, France)	Colorimetric indicator based on microbial growth, enhancing microbial safety, especially in seafood and poultry.	https://app.airsaas.io/fr/produit/cryolog, accessed on 10 September 2024
Thermochromic Labels	Timestrip^®^ (Cambridge, UK)	Labels indicate exposure to improper temperatures, ideal for real-time monitoring of chilled RTC products.	https://timestrip.com, accessed on 10 September 2024
Freshness Indicators	Fuji Seal (Osaka, Japan)	Visual indicators of freshness that extend shelf life, particularly for RTC sushi and ready-to-eat meals.	https://www.fujiseal.com, accessed on 10 September 2024
Intelligent Packaging	Mitsubishi Gas Chemical (Osaka, Japan)	Includes oxygen absorbers and moisture control agents, essential for RTC meals sensitive to oxygen and moisture.	https://us.mitsubishi-chemical.com/industry/food/, accessed on 10 September 2024
AIPIA Packaging Innovation	Sorbent Systems (Los Angeles, CA, USA)	Active packaging solutions with oxygen scavengers and moisture absorbers, maintaining freshness and safety during storage and transport.	https://www.sorbentsystems.com, accessed on 10 September 2024

## Data Availability

The original contributions presented in this study are included in the article. Further inquiries can be directed to the corresponding author.
